# Well-Being Indicators in Autistic Children and Therapy Dogs During a Group Intervention: A Pilot Study

**DOI:** 10.3390/ani15142032

**Published:** 2025-07-10

**Authors:** Viviana Orsola Giuliano, Luigi Sacchettino, Alina Simona Rusu, Davide Ciccarelli, Valentina Gazzano, Martina de Cesare, Michele Visone, Vincenzo Mizzoni, Francesco Napolitano, Danila d’Angelo

**Affiliations:** 1 Department of Veterinary Medicine and Animal Production, University of Naples Federico II, 81037 Naples, Italy; vivianaorsola.giuliano@unina.it (V.O.G.); luigi.sacchettino@unina.it (L.S.); 2 Human-Animal Interaction Research Lab, Faculty of Animal Science and Biotechnologies, University of Agricultural Sciences and Veterinary Medicine, 400372 Cluj-Napoca, Romania; alina.rusu@usamvcluj.ro; 3 Local Health Unit, 80143 Naples, Italy; davide.ciccarelli@aslnapoli1centro.it; 4 Department of Veterinary Sciences, University of Pisa, 56124 Pisa, Italy; valentina.gazzano@unipi.it; 5 Residential Centre for AAI “Coop. DogPark”, Ottaviano, 80137 Naples, Italy; info@caniledogpark.com (M.d.C.); m.visone@caniledogpark.com (M.V.); vincenzomizzoni@libero.it (V.M.); 6 CEINGE—Biotecnologie Avanzate Franco Salvatore, 80145 Naples, Italy

**Keywords:** cortisol, oxytocin, animal-assisted intervention, dog welfare, human–animal relationship, animal assisted services

## Abstract

Animal-assisted services (AAS) have been shown in multiple studies to improve a range of human psychological and physical health benefits. The aim of this pilot study is to investigate simultaneously two psycho-physiological indicators of the valence of interactions in the context of dog-assisted activities in children diagnosed with autism spectrum disorder. Ten children and four dogs experienced in AAS were involved, lasting 90 days, in weekly one-hour sessions. Before and after each session, saliva was taken in both dogs and children for determination of salivary oxytocin and cortisol levels. In addition, at the end of the program, a questionnaire was administered to both parents and dog handlers to assess the impact of AAS in children and dogs. Our results revealed no statistically significant change in cortisol and oxytocin levels in dogs enrolled throughout the sessions, while an increasing trend was noted for salivary oxytocin in 50% of the dogs and for salivary cortisol in all dogs at the end of the AAS, when compared to the pre-AAS. Salivary cortisol measurement in children with an autistic neurotype highlighted a statistically significant increase at the end of the AAS when compared to the pre-AAS, but this was not observed for oxytocin level evaluations. Regarding the perception of the children’s parents about the effects of the program, our data reported an improvement in sociability of the children in 100 percent of the cases. Furthermore, dog handlers reported an absence of signs of stress in their dogs during the sessions. Although the perceived effectiveness and quality of AAS has been demonstrated in the literature, the need to carefully select the dogs involved, considering their skills and needs, is critical to ensure their well-being in various therapeutic settings.

## Simple Summary

The role of the veterinarian expert in animal-assisted services (AAS) is crucial to ensure the well-being of animals involved in activities to support human health. The aim of this pilot study was to investigate the impact of AAS sessions on both dogs and autistic children involved, by measuring salivary levels of oxytocin and cortisol in subjects involved in the sessions. In addition, at the end of the project, the opinions about the impact of AAS by the parents of the children involved and the opinions of the dog handlers about dogs’ stress during the sessions were evaluated. Our results showed no statistically significant changes in salivary cortisol and oxytocin levels in dogs enrolled. Cortisol levels in children with an autistic neurotype highlighted a statistically significant increase, while no statistically significant change was found for salivary oxytocin. However, the perceived impact of AAS on children by the parents and on dogs by the handlers points to the efficacy of such interventions on children with an autistic neurotype, without having negative effects on the behavior and emotions of the dogs involved. This emphasizes the need to monitor the well-being of the dog–human caregiver system in AAS through complementary tools, such as biochemical markers, behavioral observations, and questionnaires on perceived efficacy and stress.

## 1. Introduction

The inclusion of companion animals in human health and welfare services has increased in recent decades, thanks to the growth of the research interest in human–animal bonds [1,2]. Several studies report that animal-assisted services (AAS) have positive effects on various aspects of human psychological and physiological health [3,4,5], such as mood improvement [6], a reduction in stress-related parameters, such as cortisol [7], a reduction in fear and anxiety [8,9,10], and the improvement of cardiovascular health [11]. Although the beneficial effects on humans have been highlighted by many researchers, attention to animal welfare during their performance in AAS is more recent and often placed within an ethical framework [12,13,14,15]. As reported by Winkle and collaborators, “Providers of animal assisted therapy have a moral and ethical obligation to extend the Do No Harm tenet to the animals with whom they work” [16]. This emphasizes the need for AAS organizations to create high quality standards through which the welfare of animals as sentient animals is ensured, as well as the achievement of therapeutic success and the sustainability of care programs [17,18,19,20,21].

In line with the international trends regarding the needs for animal welfare guidelines in the context of AAS, the Italian Ministry of Health established the ‘National Guidelines for Animal-Assisted Interventions’ in 2015 to standardize operational protocols for AAS and encouraging scientific research to produce increasingly robust data. The Italian National Guidelines provide several indications about the management of different kinds of interventions, behavioral and veterinary requirements for the animals engaged, duties that each professional figure must plays within the multidisciplinary team, and the types of training programs for AAS in which individuals participate [22]. According to Italian guidelines for canine-assisted services, the team should include the following mandatory figures: a veterinarian, qualified healthcare professional (doctor, therapist, clinical psychologist, social worker, etc.), dog trainer/handler, and program coordinator. Within this framework, animal-assisted activities are implemented through a multidisciplinary approach to best preserve the health and welfare of all individuals (including animals).

Various behavioral and physiological parameters, such as cortisol and oxytocin levels, can be useful for assessing in a non-invasive manner the welfare and the affective states of the animals included in AAS [12,17,23,24]. In fact, during stressful situations, the hypothalamic–pituitary–adrenal (HPA) axis is activated, causing the activation of the “fight-or-flight” response (i.e., increased attention and expression of stress behaviors) and the release of glucocorticoid hormones, including cortisol [17]. Cortisol can be detected in several biological matrices; in particular, saliva samples are suitable because they reflect both sympathetic nervous system (acute stress) and HPA activity [25]. In addition, saliva sampling is a non-invasive method, and the repetition of the analysis over time allows the adaptive response to be assessed even in the medium or long term. A further positive aspect of saliva sampling is that the delayed transfer from blood to saliva (20–30 min) avoids the artifacts associated with the sample collection procedure, but this procedure itself can be a stressful event for some dogs [26].

Oxytocin is often reported as an indicator of welfare or stress in dogs included in AAS. This neuropeptide is produced by the paraventricular and supraoptic nuclei of the hypothalamus and plays important roles in social behavior, including reproduction and parenting [13]. As early as the 1990s, various studies showed that oxytocin, released during positive forms of social interaction, appeared to be responsible for buffering sympathetic responses in the autonomic nervous system, such as blood pressure and heart rate; it also appeared to have antistress effects by reducing glucocorticoid stress hormones in humans and animals and it is associated with increased parasympathetic function [27]. It is therefore not surprising that more recent research has shown that positive interaction with animals stimulates the production of circulating oxytocin, which is known to have calming effects and promotes relaxation [28]. In particular, the studies on positive interactions between humans and dogs showed that blood pressure decreased significantly in both humans and dogs and that plasma oxytocin increased in both species, while plasma cortisol decreased only in humans [29,30]. Oxytocin concentrations can be quantified peripherally in plasma, urine, and saliva. Blood sampling is an invasive medical procedure that can cause stress and pain; in addition, oxytocin concentrations in the blood can vary, even within 90 s of the onset of a stimulus, and can be measured incorrectly because of binding to other molecules. Urine sampling is a non-invasive procedure, even if it presents the problem of timing, especially for testing the effect of stimuli of short duration; in addition, the significance of determining urinary oxytocin concentration is still unclear. Instead, salivary oxytocin sampling has none of these disadvantages: it is a non-invasive procedure and can be performed with specific and accurate timing (although the right timing of the oxytocin peak in saliva has not yet been precisely established) [13]. Autism spectrum disorder (ASD) includes a range of neuroevolutionary conditions characterized by differences in social competences (social cognition, communication, language, interests), and in sensory integration and regulation, as well as occurrence of repetitive and restrictive behaviors [31,32,33]. These features often generate significant barriers for individuals with autistic neurotype, preventing the meeting of their own goals in conventional social activities and exercise. Animal-assisted services offer a unique mode of nonverbal communication to take place (both multispecies and interpersonal), making it particularly suitable for this demographic category of beneficiaries [34]. Studies have shown that such interventions can improve mood, playfulness, and social interactions in children with an autistic neurotype [35], as well as language use and social interactions [36,37].

The aim of this pilot study is to investigate simultaneously two psycho-physiological indicators of the valence of interactions in the context of dog-assisted activities in a special category of beneficiaries, i.e., children with an autistic neurotype. The first objective of this pilot investigation was to assess the levels of cortisol and oxytocin salivary in both dogs and autistic children involved in group-based activities. The second objective was to evaluate the perceived impact of AAS on children’s parents and on dog handlers, and then to correlate this information with the data obtained through cortisol and oxytocin measurements.

## 2. Materials and Methods

The experimental protocols were approved by the Scientific Ethic Committee for Animal Experimentation (Centro Servizi Veterinari-University of Naples Federico II, Naples, Italy) on 4 May 2023 (Reference number: PG/2023/0051878). The informed consent of the parents or legal representative has been obtained.

### 2.1. Participants: Children and Dogs

The study included ten children with autistic neurotype and co-occurrence of symptoms of hyperactivity. The participants ranged between 6 and 12 yearsz old, consisting of nine males and one female. Specifically, the age distribution was as follows: one child was 6 years old, two were 8 years old, three were 9 years old, two were 10 years old, and one was 12 years old. The inclusion criteria were as follows: diagnosis of autism spectrum disorder confirmed by a pediatric neuropsychiatrist; absence of allergies to dogs; absence of severe motor problems preventing normal walking during sessions; ability to carry out simple instructions; absence of aggression toward animals and humans; consent from parents or legal guardian; and absence of concurrent participation in other similar studies.

All participating children were evaluated by a pediatric neuropsychiatrist and were not found to have any co-occurring conditions, such as ADHD. During the activities, a clinical psychologist—who was also involved in implementing the AAS program—observed occurrences of hyperactive behavior. These behaviors were specifically noted and used as target variables to guide the planning of subsequent activities.

The dogs involved were as follows: a Border Collie that was 2 years old, intact male; a French bulldog (FB) that was 4 years old, intact female; an 8-year-old golden retriever-like spayed male Mixed-breed (MB); and a German shepherd dog that was 3 years old, intact male. The German Shepherd (GS) has been participating in AAS programs for 3 years, the Mixed-breed for 4 years, and the Border Collie (BC) and French Bulldog for 2 years. All dogs met the behavioral and physiological requirements of the National Guidelines; in addition, they were well socialized with each other, as they had been part of previous assisted activities with adults and children.

### 2.2. Sample Collection and Cortisol—Oxytocin Measurement in Children and Dogs

All the saliva samples (both in dogs and in children) were collected at least 30 min after drinking and feeding. The sampling researcher wore a sterile mask and gloves. Salivary samples were individually collected from dogs and children by using cotton swabs (Salivette^®^ Sarstedt, AG & Co., Numbercht, Germany), before and after the first AAS (day T_0_), at the midway through the program (day T_45_), and the last meeting (day T_90_), within 15 min before and after the session by applying a saliva swab between the gums and cheeks held for 1 min. AAS were held in the afternoon, starting at 3 pm and ending at 4 pm. After the sampling, the saliva samples were placed in a polystyrene container with ice and transported to the laboratory, where they were promptly centrifuged at 3000 rpm in a centrifuge for 10 min. The samples obtained were stored at −20 °C and then processed. Cortisol and oxytocin levels were determined by immunoassay using the commercially available cortisol and oxytocin kit (FineTest, Wuhan, China) according to the manufacturer’s indications for each reference species.

### 2.3. AAS Setting and Structure of the Canine-Assisted Program

All the AAS sessions were held at a certified animal care intervention center in Naples, Italy. The sessions were conducted in a space characterized by an indoor room of about 60 square meters and an attached outdoor area of about 100 square meters.

The research lasted 90 days, with weekly meetings which were group-based; the children participated in all sessions and each child interacted with the four dogs involved during each session. All dogs were involved in the session simultaneously; each dog, in turn, performed a specific activity with each child, one by one. The activities were chosen based on the dog’s personality traits and in line with the focus of the session. Each session lasted approximately one hour. The children’s parents and all four trained handlers responsible for their dogs were present at each session. To support the welfare of the dogs during these activities, the dogs had a relaxation room with all comforts (kennel, fresh water available, chew toys, and games) and an outdoor space used for excrement before each session, and in the first minutes when the dogs were not yet involved. They received fresh water during the session, except for the last 20 min, to permit the collection of a salivary sample without any interference. At the conclusion of every session, the dogs were engaged in games or walks that met their needs and personality traits. The sessions were conducted in a setting characterized by an indoor room of about 60 square meters and an attached outdoor area of about 100 square meters. The four dogs involved were well socialized with each other, as they had undergone specific training to work in that specific setting and had participated in previous group-assisted activities with adults and children.

Activities proposed to children were divided as follows:

1st meeting:

- Opening sequence—“High Five” theme song.
- Presentation of the photograph of the four dogs.
- Live presentation of the dogs and first approach.
- Closing sequence—saying goodbye to the dogs, hand cleaning wipes, and theme song.

From the 2nd to the 6th meeting:

- Opening sequence—“High Five” theme song.
- Presentation of the activity—showing the photograph of the dogs involved.
- Activity 1 (focus on hyperactivity management)—the subject is asked to follow simple instructions to play with dogs (grab the ball, hold the ball counting to three, throw the ball to the dog, wait for the dog to return it, take the ball).
- Activity 2 (focus on sensory sphere activation)—the subject is asked to touch the dog in specific parts of the body (where is the tail? touch the ear, etc.).
- Activity 3 (focus on hyperactivity management)—the subject is asked to follow simple instructions to feed the dog (take the bowl, put the kibble in the bowl, place the bowl on the ground without dropping it, give the command to access the food).
- Activity 4 (focus on sensory sphere activation)—the subject is asked to take care of dog through epimeletic behavior, such as petting.
- Closing sequence—saying goodbye to the dogs, hand cleaning wipes, and theme song.

From the 7th to the 11th meeting:

- Opening sequence—“High Five” theme song.
- Presentation of the activity—showing the photograph of the dogs.
- Activity 1 (focus on sensory sphere activation)—the subject is asked to take care of dog through epimeletic behaviors, such as feeding, brushing and petting.
- Activity 2 (focus on sensory sphere activation)—the subject is asked to touch the olfactory mat and hide a food reward for dog. Then, the subject observes how the dog searches for food with its sense of smell and can stop to pet it.
- Activity 3 (focus on hyperactivity management)—the subject is asked to follow simple instructions to walk dog on a leash (grab the leash, hold it in your hands, follow the indicated direction, look at dog, you can ask to sit, return the leash).
- Activity 4 (focus on hyperactivity management)—the subject is asked to sit at the entrance of a tunnel, wait for the handler to position dog at the other entrance of the tunnel, say the command to make the dog cross the tunnel and welcome it with a food reward.
- Closing sequence—saying goodbye to the dogs, hand cleaning wipes, and theme song.

12th meeting:

- Opening sequence—“High Five” theme song.
- Presentation of the photograph of the dogs.
- Live presentation of the dogs and final farewell.
- Closing sequence—saying goodbye to the dogs, hand cleaning wipes, and theme song.

### 2.4. Questionnaire About the Impact of AAS on Canines and Children

The questionnaire was designed considering previous studies that investigated the perceived effectiveness of AAS by parents and medical personnel involved in the research [38,39,40]. The questionnaires, which were given to parents and handlers after the last session (T_90_) in a printed form, examined four distinct domains, namely, ANAMNESIS, EMOTIONS, BEHAVIORS, and SOCIABILITY, in both children and dogs. The items were either open questions or with multiple choice possibility. Regarding the questionnaire administered to the children’s parents, some example questions were as follows: in the domain ANAMNESIS, “Do you have animals at home?”; “Is your child on pharmacological treatment?”; “Had your child interacted with dogs before this experience?”; “If yes, how did you find the experience? Positive or Negative?”; “Had your child previously participated in similar projects involving interaction with animals?”; in the domain EMOTIONS, “Did you notice any behaviors related to the emotion of fear during the meetings?”; “Did you notice any behaviors related to the emotion of happiness during the meetings?”; “Did you notice any behaviors related to shyness during the meetings?”. In domain BEHAVIORS: “Upon returning home after the dog meetings, did you notice any changes?”, “Irritability/shouting”; “Stereotypies”; “Tendency to be alone”.

Regarding the questionnaire administered to dog handlers, some example questions were as follows: in the domain ANAMNESIS, we asked “At what age did your dog start participating in AAS projects?”; in the domain EMOTIONS “Have you noticed signs of stress (vocalizations, scratching, trembling, jumping, repetitive movements, stretching, panting, holding mouth open, licking lips, licking people or objects, self-grooming) during sessions? “; in the domain BEHAVIORS “Upon returning home after AAS meetings, did you notice an increase in: Irritation, Self-grooming”; in the domain SOCIABILITY, examples are as follows: “If you had to think about the impact of the whole course on your dog, what would you state? Increased nervousness in interaction/manipulation with children.” For a comprehensive view of the questionnaire, please see the Supplementary Materials.

### 2.5. Statistical Analysis

**Figure 1:**
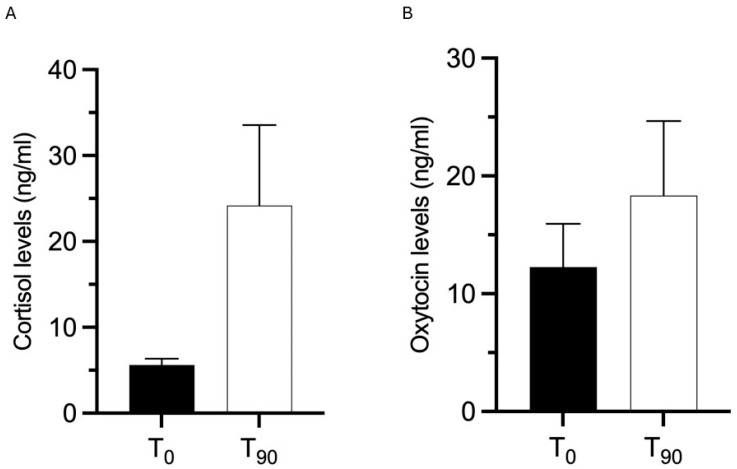
(**A**) and (**B**) display the mean salivary cortisol and oxytocin levels, respectively, for each dog at two time points: T_0_-Pre and T_90_-Post. While an increasing trend was noted for salivary oxytocin in 50% of the dogs and for salivary cortisol in all dogs, these changes were not statistically significant when comparing T_0_-Pre and T_90_-Post measurements. Data are presented as mean ± SEM.

**Figure 2:**
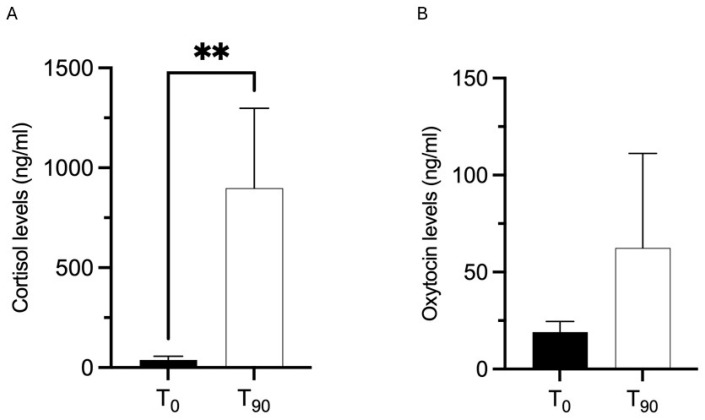
Mean cortisol (**A**) and oxytocin (**B**) levels in the autistic children, at T_0_-Pre and T_90_-Post. A statistically significant change was observed in CORT levels between the T_0_-Pre and T_90_-Post, not observed for OT level evaluations. All values are expressed as mean ± SEM. ** *p* < 0.01.

The mean cortisol and oxytocin levels in dogs (Table 1) were analyzed using an ordinary one-way ANOVA, followed by multiple comparisons, when required, and assuming the overall Gaussian distribution of data, as assessed by Shapiro–Wilk test. Data of cortisol and oxytocin levels shown in Figure 1 were analyzed using a paired *t*-Test. On the other hand, mean cortisol and oxytocin levels in autistic children (Table 2) were analyzed using a Kruskal–Wallis test, since we could not assume the normal distribution of the values, as assessed by Shapiro–Wilk test. Therefore, data of cortisol and oxytocin levels shown in Figure 2 were analyzed using a Wilcoxon test.

Data were analyzed through Wilcoxon matched-pairs signed-rank test (La Jolla, San Diego, CA, USA). Results were considered statistically significant for *p* value > 0.05.

## 3. Results

### 3.1. Evaluation of Cortisol and Oxytocin Levels in Both Pets and Autistic Children During the AAS

Here, we analyzed the potential effect of AAS upon salivary cortisol (CORT) and oxytocin (OT) levels during the 90-day-program between pre- and post-sessions, in each subject involved in the AAS. With this aim, we collected saliva samples at three different time points, namely at the beginning (T_0_), mid-term (T_45_) and at the end of the AAS (T_90_). We did not observe any significant effect on CORT and OT mean levels (average ng/mL ± SEM) in dogs enrolled throughout the sessions (CORT: FB = 94.68 ± 81.25; GS = 14.18 ± 5.996; BC = 13.15 ± 2.266; MB = 8.317 ± 2.831; one-way ANOVA, F_(3, 20)_ = 1.035, *p* = 0.3985; OT: FB = 52.50 ± 30.46; GS = 13.28 ± 3.486; BC = 14.16 ± 4.252; MB = 10.13 ± 1.999; one-way ANOVA, F_(3, 20)_ = 1.674, *p* = 0.2047, Table 1).

Then, we moved towards the evaluations of both CORT and OT mean levels in the saliva of autistic children. Differently to what was observed in the dogs, data from autistic children did not follow a normal distribution, as assessed by Shapiro–Wilk test, so we performed nonparametric tests. In this respect, we did not find any significant effect of CORT levels in our autistic children over the analyzed time points (1-AFP = 6.203 ± 2.119; 2-DSD = 499.2 ± 430.9; 3-GR = 38.31 ± 20.85; 4-NF = 780.9 ± 534.5; 5-PG = 299.8 ± 250.2; 6-RS = 6323 ± 6319; 7-SJ = 940.0 ± 845.3; 8-SD = 39.55 ±26.41; 9-ZA = 342.6 ± 310.5; 10-MSF = 60.92 ± 32.64; Kruskal–Wallis test: *p* = 0.2084). On the other hand, a main effect of OT levels was documented (1-AFP = 3.290 ± 1.453; 2-DSD = 30.52 ± 15.96; 3-GR = 25.44 ± 11.34; 4-NF = 163.8 ± 71.68; 5-PG = 35.43 ± 23.90; 6-RS = 9.792 ± 4.374; 7-SJ = 42.03 ± 21.93; 8-SD = 12.41 ± 5.363; 9-ZA = 13.81 ± 6.587; 10-MSF = 21.12 ± 11.10; *p* = 0.0429), as following multiple comparison remarked that OT levels of 1-AFP subject were different from those of 4-NF (*p* = 0.0033). See Table 2.

### 3.2. Parental Opinions About the Impact of AAS on Autistic Children

**Figure 3:**
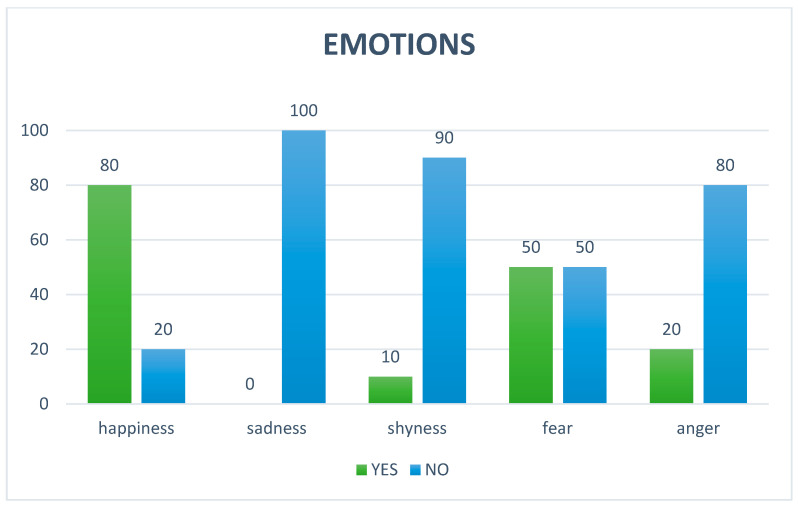
“Emotions” domain of the questionnaire administered to the children’s parents. On the *x*-axis are listed the emotions expressed by the children during the AAS sessions. On the *y*-axis, the responses given by the parents, expressed in percentages.

**Figure 4:**
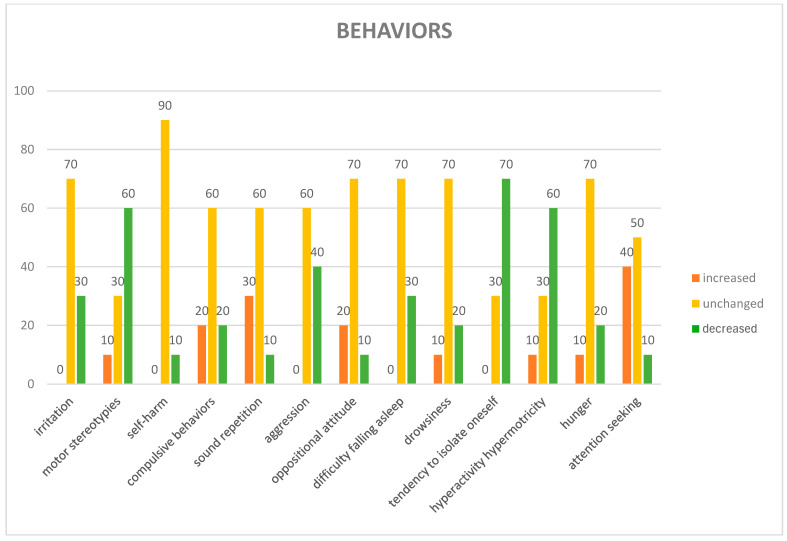
“Behaviors” domain of the questionnaire administered to children parents. On the *x*-axis are listed the variation in children’s behaviors noticed by their parents after the AAS sessions. On the *y*-axis, the responses given by the parents, expressed in percentages.

**Figure 5:**
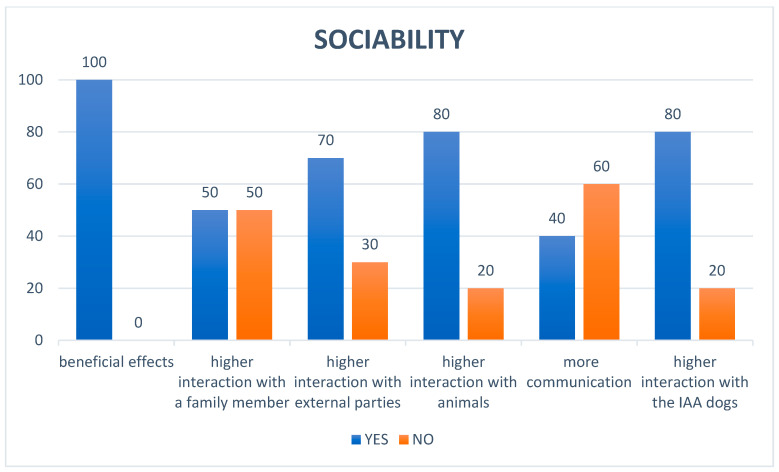
“Sociability” domain of the questionnaire administered to the children’s parents.

Regarding the questionnaires administered to all parents of children involved in AAS at T_90_, the detailed anamnesis of the participants revealed specific characteristics of the sample involved in the study: It emerged that none of the participating children (100%) had pets at home; a significant portion of the sample, 60%, had previous interactions with dogs, although not owned. Of these prior experiences, the majority (four individuals) were perceived as positive, while a smaller number (two individuals) reported a negative interaction. Regarding participation in structured programs, the detailed anamnesis showed that most of the children (80%) had never participated in AAS prior to this experience. It was found that only a small percentage of participants, 10%, were under pharmacological treatment. In the Emotion domain, happiness is the dominant emotion, apart from fear, which is manifested in certain subjects (Figure 3). In the Behavior domain, many behaviors tended to remain unchanged for irritation compulsive behaviors: for 70% and 90% of cases, respectively, no change was noticed. For difficulty falling asleep and tendency to isolate oneself, repetition of sound or sleepiness, a balance was noted, a prevalence of “unchanged” (both at 70%), indicating that for most of the people observed, these habits remained constant. Motor stereotypies showed a decrease of 60%, oppositional attitude showed a decrease of 40%, attention-seeking showed an increase in 40% of cases, self-injury and hyperactivity/hypermotricity also showed a decrease of 40%; while showing an overall decrease for a portion of the cases, there was still an overall increase in 30% of cases (Figure 4). In the Sociability domain, the beneficial effects were perceived by all, with 100% “YES”. Interaction with family members was equally divided between “YES” and “NO” (50%). There was more interaction with outside people (70%) and with animals and AAS dogs (both 80%) (Figure 5).

On the *x*-axis are listed the effects of the AAS program on children’s behaviors about the sociability noticed by their parents, once they were home. On the *y*-axis are the responses given by the parents, expressed in percentages.

### 3.3. Opinions of the Dog Handlers About the Impact of AAS on Dogs

All four dog handlers completed questionnaires, in which the items were organized in four domains. Regarding the first domain, the “ANAMNESIS”, the dog handlers reported that three of the dogs were adopted at two months of age, and began performing AAS at two years of age, while the mixed-breed was adopted at two years of age by the shelter and began participating in AAS projects at three years of age. Regarding the “EMOTIONS” domain, all four dog handlers reported that they did not observe manifestations of emotions such as fear, anger, sadness, and shyness by their dogs during the sessions, while three out of four highlighted the manifestation of the emotion happiness. Regarding the “BEHAVIORS” domain, all dog handlers reported that when they returned home after each session, they did not record an increase in the following behaviors of their dogs: irritation, self-grooming, scratching, vocalizations, tremors, stereotypies, reactivity, sleep disturbances, drowsiness, isolation tendency, restlessness, or hunger. Regarding the “SOCIABILITY” domain, all dog handlers reported that they do not consider that the AAS program had a negative impact on their dogs’ sociability, defined as interaction/manipulation by children, adult strangers, familiar people, and other dogs, nor was there any nervousness or avoidance in noisy and crowded places.

## 4. Discussion

This pilot study investigated oxytocin and salivary cortisol levels in both dogs and autistic children during a group-based AAS sessions, seeking to provide additional data to the current literature. Despite the rapid growth of research evidence supporting the effectiveness of AAS in various treatment plans of ASD [41,42,43], current findings remain fragmented due to various factors such as experimental design, sample selection, outcome measurement, and lack of longitudinal follow-up. In addition, to our knowledge there are no studies that simultaneously investigate the welfare of the participant and the animal involved using biological markers [44]. These limitations have hampered the comprehensive compilation of scientific evidence supporting the effectiveness of these interventions, thus limiting their wider dissemination and implementation [45].

Regarding cortisol levels, we did not find any significant effect of cortisol levels in pre- and post-sessions in autistic children, while statistically significant change was observed in cortisol levels between the T_0_-Pre and T_90_-Post. The interpretation of these data requires caution. To begin with, previous studies in autistic children have not shown a clear pattern of cortisol stress response, along with higher levels of emotional stress than in the general population, making individuals more vulnerable to the development of altered HPA axis function [46]. In addition, factors such as previous experiences with dogs or specific dynamic interaction may have played a role in children’s perceptions, since a significant portion of the sample (60%) have had previous interactions with dogs. Of these previous experiences, most (four individuals) were perceived as positive, while a smaller number (two individuals) reported a negative interaction. Furthermore, it should be kept in mind that our results showed that most of the children (80% of the sample) had never participated in AAS before this experience; this indicates that for most of the participants, this AAS program represented their first exposure to this type of social event. Thus, our findings could be interpreted in accordance with previous studies that have reported higher cortisol levels in autistic neurotype populations, in response to a variety of novel stimuli and situations [47,48]. In conclusion, the increase in cortisol could be related to physical activity or arousal—since in several sessions the children were involved in motor activities—and not necessarily to psychological stress.

Regarding the canine cortisol level, our results observed no significant effect of cortisol in the enrolled dogs during pre- and post-sessions; however, at the end of the program (T_90_-Post) we noticed a trend (although not significant) of increasing cortisol levels in the enrolled dogs when compared with the T_0_-Pre time point. Several factors have been reported to influence basal salivary cortisol levels, such as site of residence, animal size, and sterilization conditions [49]. In contrast, other sources of biological variability (such as the age and sex of the animal, as well as the time of day and place of collection) have been reported to have no influence on salivary cortisol concentrations [50,51]. In fact, previous research on dogs involved in AAS has already revealed an increase in salivary cortisol following the sessions [52]. In particular, Haubenhofer and Kirchengast [53] had reported that salivary cortisol levels of therapy dogs were significantly higher during therapy days than during control days, results confirmed by a further article [54] that has showed a significant increase in salivary cortisol levels in therapy dogs between the start and 1 h after an AAS session. Even though these studies show that therapy work causes a sudden rise in cortisol levels and that this activity is physiologically arousing, it is unclear whether this rise is caused by stimulating interactions with an unknown person, or living in an unfamiliar environment, or a combination of these factors and/or other factors [52]. In addition, it is important to consider that other factors related to the tasks performed (e.g., the use of food rewards or ball games) might have influenced the dogs’ levels of arousal or frustration during the study. Although the dogs were already familiar with each other and the environment, these emotional states may have had an impact on cortisol measurements. Therefore, it is essential to consider breed and specific behavioral and personality traits of the dogs that are included in AAS. Further research that takes these factors into account could provide a deeper understanding of the hormonal and behavioral responses of dogs during these interactions. Ultimately, the increase in cortisol found in both dogs and children involved could also reflect hormonal synchronization between humans and dogs, a phenomenon observed in previous research [55]. Indeed, assisted activities should be interpreted as two-way experiences, in which both dogs and children experience shared emotions and feelings in an inter-species context.

Regarding oxytocin levels, the results showed a tendency of fluctuating levels before and after the session, at all three sampling times of the program; however, we found an increased trend, not statistically significant, in four children involved at T_90_-Post compared with T_0_-Pre, and a concomitant rise, not statistically significant, in salivary oxytocin in two dogs involved at T_90_-Post compared with T_0_-Pre. These variabilities in oxytocin responses might suggest individual differences in sensitivity to AAS. The children in our study exhibited a variety of expressions of self-regulating behaviors, which might have been reflected in their interactions with the animals. For example, some of them had more difficulty touching the animal, or following the dog handler’s instructions, some were more impetuous, and some were more easily distracted than others. This diversity of behavioral displays makes it more challenging to isolate the specific impact of AAS in this category of beneficiaries.

Additionally, the emotional history of human–animal interactions can shape how animals respond. Previous experiences can influence their reactions in future encounters, emphasizing the importance of considering the entire relationship between the child and the animal [56]. The results suggest the need to personalize AAS interventions based on the individual characteristics of children, considering their fears, comorbidities, and attachment style. It is also crucial to investigate more closely whether dogs can gain or suffer from interactions with the beneficiaries [18,57]. Another potential effect not to be overlooked is the timing of saliva collection, since its precise timing and duration of salivary oxytocin peaks in dogs remain unclear [13]. This warrants further investigation to refine our understanding of oxytocin’s role in AAS. Our findings highlight the complexity of hormonal responses during AAS. Future studies should explore individual differences and interaction characteristics to better understand hormonal changes in dogs included in AAS. Another factor that might influence the ways the dogs are interacting with groups of children is the sex composition of the group. In our study, the sex-biased composition of our participant sample resulted from convenience sampling. Additionally, it is well documented that males are diagnosed with ASD at a rate approximately three times higher than females. While research shows that both autistic boys and girls benefit from interactions with animals, there is currently no evidence of sex-based differences in their play styles or interactions with animals during AAS. However, drawing on established findings from developmental psychology, it can be inferred that boys may be more likely to engage in direct interactions with dogs, such as rough-and-tumble play, whereas girls may be more inclined toward petting and verbal communication [58]. Our data in dogs appear to contradict the ones in humans, where oxytocin and cortisol often exhibit an inverse relationship [29]. The dogs in our study showed a trend of higher levels of cortisol salivary, which was consistent with the conclusions of Marinelli and colleagues, who highlight more signs of canine stress during interaction with children under 12 [23]. This is crucial in our context, as our study involved children under 12, whose behaviors can be unpredictable and unexpected for dogs. A further negative stressor could be the display of stimming behaviors for self-regulation of autistic children [33], such as repetitive behavior, high-pitched vocalizations etc., which can pose difficulties in the process of interspecific communication with dogs. In fact, previous studies have reported the negative impact on the well-being of dogs due to stressors present during AAS, such as noisy or physically uncontrolled users, interactions with other humans or animals and unfamiliar environments, sudden noises, unexpected events with children, and limited space [23,59]. Therefore, the increase in cortisol could be interpreted as a response to stress in this group therapy context. A further interpretation of the increase in cortisol could depend on an individual perception of the stimulus, past experiences, and genetics, as reported by studies of Haubenhofer and Kirchengast [60]. However, in analyzing our data, we cannot overlook the fact that recent studies on canines also report an increase in cortisol following positive human–animal interactions [61]; in fact, as suggested by Chmelíková and coworkers, increased cortisol is not only related to stressful situations, but is also positively associated with improvements in cognitive and emotional processes [62], activated during AAS. To note, we are aware that this is a pilot investigation based on a small sample size and without a control condition, such as children interacting only with the handlers versus children interacting with the handlers and the dogs.

Regarding the perceptions of the children’s parents on the effects of the program, our data showed improvement for some behaviors and worsening or unchanged behavior for others. The emotion present was most represented by happiness. The greatest improvement can be seen in sociability, with the overall perception of benefit confirmed by 100% of the parents. However, these data should be evaluated with caution, since, as reported by Van der Steen and collaborator [63], parents of children who have participated in AAS are more likely to report positive results, expecting a positive impact on child behavior; indeed, such bias is well described by social science researchers [64,65]. In our view, this potential efficacy comes from the group-based approach, as parents may have perceived improvements in their children due to social time, with dogs (multispecies interactions), and with other children (interpersonal interactions). Nevertheless, our results revealed that the perception of the effectiveness of the AAS program on children went in the same direction as regarding the effects on dogs, as dog handlers reported an absence of signs of stress in their dogs during the sessions. Even if the perceptions of AAS quality are generally favorable, and these practices are often considered non-stressful for the participating dogs, acute stress, burn out, and adverse interactions cannot be overlooked [59]. In the literature, there have been reported instances of inappropriate behaviors, such as teasing and mistreatment of therapy dogs, indicating the need for careful monitoring and management of interactions [66]. Furthermore, cases where the dogs included in treatments have exhibited signs of stress, such as excessive panting and fatigue, necessitating the premature termination of studies, underscore the potential for AAS to impact animal health negatively [67]. It is necessary to continue to pose questions on the ethical level as well, as reported by Contalbrigo and collaborators in a recent article, in which they call attention to the need for an approach that is balanced and responsible toward each individual dog involved in AAS [68]. Interestingly, our pilot study seems to indicate that signs of stress were limited during the sessions; this could be since the dogs were specifically trained to operate in this setting and had previously participated in AAS group sessions with children and adults. Therefore, the perceptions of dog handlers may have been influenced by this, as they reported no negative impact on their dogs in the questionnaires [69]. Recognizing these concerns, organizations like the International Association of Human–Animal Interaction Organizations (IAHAIO) have published guidelines to minimize work-related stress and enhance the quality of life for therapy animals [19,70]. Additionally, initiatives in countries like Austria have led to the implementation of legal regulations for therapy dog certification, including regular health and temperament assessments [12]. In this, a key role can be played by the veterinarian expert in AAS, who in science and conscience is adequately trained to ensure the welfare of animals involved in activities supporting human health. In fact, in accordance with the Italian guidelines for AAS, the veterinary experts in AAS collaborates with the dog handler to ensure the animal’s well-being by assisting in the selection of the species and individual to be involved in AAS, as well as directing the appropriate management of the animal in the operational setting. Despite these efforts, the diverse nature of AAS and the lack of universal standardization continue to pose challenges in ensuring consistent animal welfare.

## 5. Conclusions

Our results highlight the complexity of hormonal responses during AAS, as revealed by simultaneous analysis of salivary cortisol and oxytocin levels in children with an autistic neurotype and dogs involved in group AAS. The impact of AAS on children with an autistic neurotype was perceived as positive, without having perceived negative effects on the behavior and emotions of the dogs involved. However, this underscores the need to carefully select the dogs involved, considering their personality traits and monitoring their well-being. Future studies are needed to investigate the role of oxytocin and cortisol in dogs and children during individual AAS sessions.

## 6. Limitations

We consider that it is important to acknowledge certain limitations. To begin with, we are aware that this is a pilot investigation based on a small sample size and without a control condition, such as children interacting only with the handlers versus children interacting with the handlers and the dogs. In addition, a further limitation is the recall bias; children’s parents and dog handlers had to remember events and behaviors that occurred during the entire project. Another limitation is due to dog handler assessments, which may be subject to bias because of their direct professional involvement and potential financial interest in the sessions. These were compromises that we felt were worthwhile, given the ability to collect data on the effectiveness of AAS perceived by parents of children with an autistic neurotype and involved dog handlers, limiting the value of the data. However, a strength of our pilot study is that we investigated salivary cortisol and oxytocin levels in all subjects involved in AAS, thus attempting to provide new data to the current literature.

## Figures and Tables

**Table 1:** Mean salivary levels of cortisol and oxytocin pre- and post-sessions in the enrolled dogs. No statistically significant change was observed in CORT and OT levels in dogs enrolled throughout the sessions.

CORT Levels (ng/mL) at Different Time Points						
ID	T_0_-Pre	T_0_-Post	T_45_-Pre	T_45_-Post	T_90_-Pre	T_90_-Post
French Bulldog (FB)	3.46	0.73	9.53	499.13	4.69	50.54
German Shepherd (GS)	5.96	43.60	13.39	9.21	5.95	6.94
Border Collie (BC)	6.28	15.90	14.98	18.67	6.04	17.01
Mixed-breed (MB)	6.76	6.28	6.07	3.93	4.56	22.30
**OT levels (ng/mL) at Different Time Points**						
**ID**	**T_0_-Pre**	**T_0_-Post**	**T_45_-Pre**	**T_45_-Post**	**T_90_-Pre**	**T_90_-Post**
French Bulldog (FB)	3.36	74.30	3.61	193.53	3.37	36.80
German Shepherd (GS)	20.91	5.40	17.57	3.51	23.70	8.61
Border Collie (BC)	10.47	15.68	5.30	33.00	4.66	15.84
Mixed-breed (MB)	14.35	16.20	8.23	6.32	3.56	12.10

Similarly, at the end of the program (T_90_-Post) paired Student *t*-test showed a trend (although not significant) towards an increase in both CORT and OT levels in the enrolled dogs, when compared with T_0_-Pre time point (CORT: *p* = 0.1592; OT: *p* = 0.5804; Figure 1).

**Table 2:** Salivary levels of cortisol and oxytocin pre- and post-sessions in autistic children. Our results did not find any significant effect on CORT levels in our autistic children over the analyzed time points; while an increased trend of CORT was found, it was not statistically significant in the children involved at T_90_-Post compared with T_0_-Pre.

CORT Levels (ng/mL) at Different Time Points						
ID	T_0_-Pre	T_0_-Post	T_45_-Pre	T_45_-Post	T_90_-Pre	T_90_-Post
1-AFP	3.50	3.51	3.52	16.63	5.87	4.19
2-DSD	197.50	3.51	3.50	115.68	26.68	2648.22
3-GR	9.63	4.38	3.86	114.96	4.97	92.06
4-NF	5.54	27.70	479.60	581.31	181.25	3409.70
5-PG	69.96	4.31	3.69	11.33	1544.25	165.08
6-RS	3.57	4.96	3.50	4.41	37,918.89	4.95
7-SJ	23.40	41.72	3.49	23.91	5155.83	391.55
8-SD	14.64	6.80	3.50	33.68	8.91	169.77
9-ZA	36.54	112.05	5.66	4.59	3.91	1892.92
10-MSF	3.60	18.20	3.61	26.77	111.50	201.81
**OT Levels (ng/mL) at Different Time Points**						
**ID**	**T_0_-Pre**	**T_0_-Post**	**T_45_-Pre**	**T_45_-Post**	**T_90_-Pre**	**T_90_-Post**
1-AFP	1.09	1.25	10.38	3.29	1.89	1.84
2-DSD	62.46	95.14	1.12	14.11	8.44	1.87
3-GR	11.02	21.59	72.05	44.21	1.86	1.92
4-NF	10.05	65.64	111.50	88.40	211.64	495.70
5-PG	28.77	20.21	7.58	1.98	151.46	2.59
6-RS	14.30	6.64	4.18	1.96	29.51	1.92
7-SJ	17.76	25.56	0.42	142.12	62.01	4.32
8-SD	20.62	7.73	1.98	6.43	2.34	35.38
9-ZA	21.37	43.28	8.73	1.93	1.85	5.69
10-MSF	2.91	13.80	6.57	26.18	3.55	73.72

Salivary cortisol (ng/mL) measurement in autistic children showed a statistically significant increase at the end of the program (T_90_-Post), when compared to the T_0_-Pre (Wilcoxon test, *p* = 0.0020), not observed for OT level evaluations (*p* = 0.8457) (Figure 2).

## Data Availability

The original contributions presented in this study are included in the article/supplementary material. Further inquiries can be directed to the corresponding authors.

## Supplementary Materials

The following supporting information can be downloaded at: https://www.mdpi.com/article/10.3390/ani15142032/s1, Questionnaire about Impact of AAI on Canine and Children.
