# Supplementary material for: Well-Being Indicators in Autistic Children and Therapy Dogs During a Group Intervention: A Pilot Study
Source: Animals (Basel). 2025 Jul 10;15(14):2032. doi: 10.3390/ani15142032 (PMC12291636; doi:10.3390/ani15142032)
Supplement: Supplementary file 1 [file animals-15-02032-s001.zip › animals-3700749-supplementary.pdf]

Questionnaire about Impact of AAI on Canine and Children

Parents version

ANAMNESIS

- Pharmacological treatment YES NO
- Had your child interacted with dogs before this experience? YES NO
- Had your child previously participated in similar projects involving interaction with animals? YES NO
    - If yes, how did you find the experience? Positive Negative

EMOTIONS

- Did you notice any behaviors related to the emotion of fear during the meetings? YES NO
- Did you notice any behaviors related to the emotion of happiness during the meetings? YES NO
- Did you notice any behaviors related to shyness during the meetings? YES NO
- Did you notice any behaviors related to the emotion of anger during the meetings? YES NO
- Did you notice any behaviors related to the emotion of sadness during the meetings? YES NO

BEHAVIORS

- Upon returning home after the dog meetings, did you notice any changes in your child?
    - Irritability / shouting DECREASED INCREASED UNCHANGED
    - Self-harm DECREASED INCREASED UNCHANGED
    - Stereotypies (motor) DECREASED INCREASED UNCHANGED
    - Compulsive and ritualistic behaviors DECREASED INCREASED UNCHANGED
    - Repetition of sounds, words, or phrases heard DECREASED INCREASED UNCHANGED
    - Aggressiveness DECREASED INCREASED UNCHANGED
    - Oppositional attitude DECREASED INCREASED UNCHANGED
    - Difficulty falling asleep / Insomnia DECREASED INCREASED UNCHANGED
    - Drowsiness DECREASED INCREASED UNCHANGED
    - Tendency to be alone DECREASED INCREASED UNCHANGED
    - Hyperactivity / hypermotricity DECREASED INCREASED UNCHANGED
    - Hunger DECREASED INCREASED UNCHANGED
    - Seeking attention DECREASED INCREASED UNCHANGED
    - Seeking physical contact with a family member DECREASED INCREASED UNCHANGED

SOCIABILITY/INTERACTION

- If you had to consider the effects of your child's participation in these meetings, what would you say?

  - Beneficial effect YES NO
  - Increased socialization or interaction towards a family member YES NO
  - Increased socialization and interaction with people outside the family unit YES NO
  - Increased socialization and interaction with animals in everyday life YES NO
  - Increased communication in everyday life YES NO
  - Increased interaction / communication with dogs, meetings after meeting YES NO

Dog handler version

Name of the dog

Age of the dog

Sex

Sterilised/castrated Yes NO

Breed

Place of adoption

Age of adoption

Context of life

At what age did your dog start participating in IAA projects?

On average, during the sessions how participatory was he?

Very much quite poco for nothing

Have you noticed signs of stress (vocalizations, scratching, tremors, jumps, repeated movements, stretching; Wheezing, keeping your mouth open, licking your lips, licking people or objects, self-grooming) during sessions? Yes NO

Did you notice any fear-related behaviors during the sessions? Yes NO

Have you noticed any behaviour related to the emotion happiness during the sessions? Yes NO

Have you noticed behaviors related to timidity during the sessions? Yes NO

Did you notice any anger-related behavior during the sessions? Yes NO

Did you notice any behaviour related to the emotion sadness during the sessions? Yes NO

<u>On your way home</u>, after the IAA meetings, you noticed an increase in:

Irritation Yes NO

Self-grooming Yes NO

Scratch Yes NO

Vocalizations Yes NO

tremors Yes NO

Stereotypie Yes NO

Reactivity Yes NO

Sleep disorders (e.g. insomnia) Yes NO

Drowsiness Yes NO

Tendency to isolate oneself Yes NO

Restlessness Yes NO

Hunger	Yes	NO

If you were to think about the impact of the entire route on your dog, what would you say?

Increased nervousness in interaction/manipulation with children	Yes	NO
Greater avoidance of interaction/manipulation with children	Yes	NO
Increased nervousness in interaction/manipulation with an alien adult	Yes	NO
Greater avoidance in interaction/manipulation with an alien adult	Yes	NO
Increased nervousness in interaction/manipulation towards a family member	Yes	NO
Increased avoidance in interaction/manipulation towards a family member	Yes	NO
Increased nervousness in interaction with other dogs	Yes	NO
Increased avoidance in interaction with other dogs	Yes	NO
Increased nervousness in noisy and crowded places	Yes	NO
Increased avoidance in noisy and crowded places	Yes	NO

I have not noticed any deleterious effect on my dog ☐
